# Flux-tunable heat sink for quantum electric circuits

**DOI:** 10.1038/s41598-018-24449-1

**Published:** 2018-04-20

**Authors:** M. Partanen, K. Y. Tan, S. Masuda, J. Govenius, R. E. Lake, M. Jenei, L. Grönberg, J. Hassel, S. Simbierowicz, V. Vesterinen, J. Tuorila, T. Ala-Nissila, M. Möttönen

**Affiliations:** 1 QCD Labs, QTF Centre of Excellence, Department of Applied Physics, Aalto University, P.O. Box 13500, FI-00076 Aalto, Finland; 2 National Institute of Standards and Technology, Boulder, Colorado, 80305 USA; 3 VTT Technical Research Centre of Finland Ltd, P.O. Box 1000, FI-02044 VTT, Finland; 4 MSP group, QTF Centre of Excellence, Department of Applied Physics, Aalto University, P.O. Box 13500, FI-00076 Aalto, Finland; 5 Nano and Molecular Systems Research Unit, University of Oulu, P.O. Box 3000, FI-90014 Oulu, Finland; 6 Departments of Mathematical Sciences and Physics, Loughborough University, Loughborough, Leicestershire LE11 3TU United Kingdom; 7 Department of Physics, Brown University, Box 1843, Providence, Rhode Island 02912-1843 USA

## Abstract

Superconducting microwave circuits show great potential for practical quantum technological applications such as quantum information processing. However, fast and on-demand initialization of the quantum degrees of freedom in these devices remains a challenge. Here, we experimentally implement a tunable heat sink that is potentially suitable for the initialization of superconducting qubits. Our device consists of two coupled resonators. The first resonator has a high quality factor and a fixed frequency whereas the second resonator is designed to have a low quality factor and a tunable resonance frequency. We engineer the low quality factor using an on-chip resistor and the frequency tunability using a superconducting quantum interference device. When the two resonators are in resonance, the photons in the high-quality resonator can be efficiently dissipated. We show that the corresponding loaded quality factor can be tuned from above 10^5^ down to a few thousand at 10 GHz in good quantitative agreement with our theoretical model.

## Introduction

One of the most promising approaches to building a quantum computer is based on superconducting qubits in the framework of circuit quantum electrodynamics^1–6^. However, not all of the criteria for a functional quantum computer^7^ have been achieved simultaneously at the desired level. In particular, computational errors need to be mitigated with quantum error correction^8,9^. Many quantum error correction codes require frequent initialization of ancillary qubits during the computation. Thus, fast and accurate qubit reset is a typical requirement in the efficient implementation of quantum algorithms. To date, several approaches for qubit initialization have been studied^10–14^. Initialization to the ground state by waiting is a straightforward method but it becomes impractical in repeated measurements of qubits with long lifetimes. Therefore, active initialization is advantageous. Furthermore, it may be beneficial to design individual circuits for qubit control, readout, and initialization in order to avoid performance-limiting compromises in the optimization of the circuit parameters. In this work we focus on a specialized initialization circuit, which remains to be implemented in superconducting quantum processors.

Recently, a promising qubit initialization protocol based on dissipative environments was proposed in refs^15,16^. In this proposal, a resistor coupled to a frequency-tunable resonator quickly absorbs the excitation from the qubit when tuned in resonance. In this paper, we experimentally realize such a tunable dissipative environment and study its effect on a superconducting resonator. Tunable superconducting resonators have been demonstrated previously^17–22^ but without engineered dissipation arising from on-chip normal-metal components. In addition to quantum computing, very sensitive cryogenic detectors^23–25^ may benefit from tunable dissipation for calibration purposes. Furthermore, tunable transmission lines are also useful in studying fundamental quantum phenomena^26,27^.

Although dissipation is in some cases beneficial for quantum computing^28^, lossy materials are typically harmful for qubit lifetimes during computation. Therefore, one needs to be able to switch the dissipation on and off deterministically. In state-of-the-art experiments, quality factors, *Q*, above 10^6^ indicating very low dissipation have been achieved with coplanar-waveguide resonators^29^. Various materials and methods have been studied for fabricating high-*Q* resonators^30–33^. Here we fabricate high-*Q* resonators based on niobium on a silicon wafer. In addition, we tune the *Q* factor from above 10^5^ down to a few thousand by coupling the resonator relatively strongly to a dissipative element. Importantly, the integrated resistive element we introduce does not inherently degrade the *Q* factor when it is weakly coupled to the resonator compared to similarly fabricated resonators without any engineered resistive elements.

## Results

### Experimental samples

**Figure 1:**
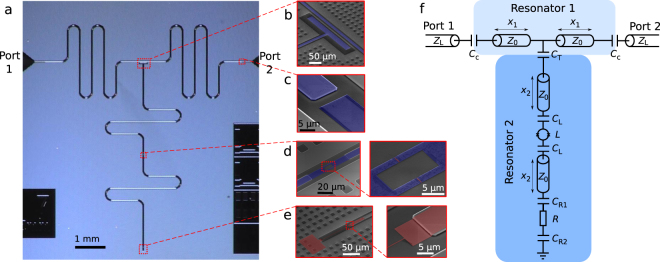
Sample structure. (**a**) Optical top image of the measured sample. (**b**) False-colour scanning electron microscope image of the coupling capacitor between the two resonators, and (**c**) between Resonator 1 (light blue) and the port to the external transmission line (dark blue). (**d**) Two micrographs of the SQUID loop highlighted in blue and the junctions highlighted in red. (**e**) Two micrographs of the termination Cu resistor (red). (**f**) Electrical circuit diagram of the sample. Resonators 1 and 2 with characteristic impedances *Z*_0_ are coupled to each other by a coupling capacitance *C*_T_ and to external transmission lines with characteristic impedance *Z*_L_ by capacitances *C*_C_. The inductance of the SQUID is denoted by *L*, and the termination resistance by *R*. The SQUID is connected to the centre conductor of Resonator 2 line with capacitances *C*_L_, and the resistor to centre conductor and ground with *C*_R1_ and *C*_R2_, respectively. The lengths of the resonator sections are denoted by *x*_1/2_. The image in panel (**a**) is from Sample A, and those in panels (**b**)–(**e**) from Sample B.

The structure of our device is presented in Fig. 1 together with the corresponding electrical circuit diagram which defines the symbols used below. The device consists of two coupled resonators, Resonator 1 with a fundamental frequency of 2.5 GHz, and Resonator 2 with a tunable frequency. Both ends of Resonator 1 couple capacitively (*C*_C_) to external circuitry for scattering parameter measurements. The even harmonics of Resonator 1 interact with Resonator 2 since there is a voltage antinode at the center of the half-wave Resonator 1, and hence, the capacitive (*C*_T_) coupling to Resonator 2 is significant.

The resonators are fabricated out of niobium in a coplanar-waveguide geometry. The modes of Resonator 2 are tunable owing to a superconducting quantum interference device (SQUID), acting as a flux-tunable inductance, placed in the middle of the resonator. The SQUID is integrated into the center pin of the waveguide and consists of two aluminium layers separated by an insulating aluminium oxide layer. When the resonance frequencies of the two resonators meet, we expect a degradation of the Resonator 1 quality factor because Resonator 2 is terminated with a dissipative on-chip resistor (*R* = 375 Ω) made of copper. Importantly, the device is designed to retain a high quality factor of Resonator 1 whenever Resonator 2 is far detuned.

We study two samples, Sample A and B, which are nominally identical, except for the length of Resonator 2. We mostly focus on Sample A which has a wider tuning range of the quality factor of Resonator 1. The samples are measured at a cryostat temperature of approximately 10 mK. The theoretical model described in Methods reveals all the essential features of the two samples with a single set of parameters given in Table 1. See Methods for the details of the sample fabrication.

**Table 1:** Simulation parameters.

Parameter	*C*_C_ (fF)	*C*_T_ (fF)	*C*_L_ (pF)	*C*_R1_ (pF)	*C*_R2_ (pF)	*C*_l_ (pF/m)	*R* (Ω)	*Z*_0_ (Ω)	*Z*_L_ (Ω)	*ε*_eff_	*x*_1_ (mm)	*x*_2_ (mm)	*Q*_int,1_	*I*_0_ (nA)
Value	1	5	2.8	4.0	28	180	375	50	50	6.35	12	7.5 (8.0)	1 × 10^5^	255

See Fig. 1f and text for the definition of the symbols. Samples A and B have the same parameter values except for the length *x*_2_, where the value for Sample B is given in parenthesis.

### Flux dependence of the resonance frequencies

**Figure 2:**
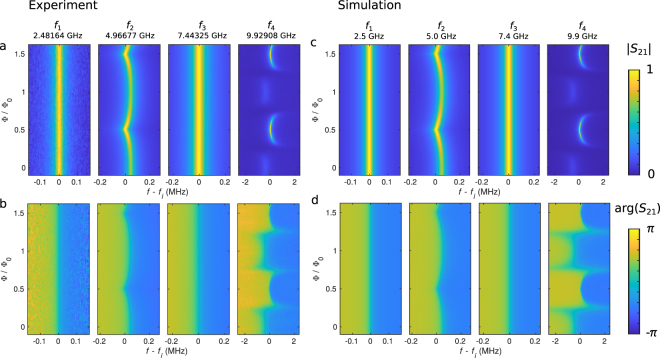
Resonances of Sample A. (**a**,**b**) Experimental and (**c**,**d**) computational (**a**,**c**) amplitude and (**b**,**d**) phase of the scattering parameter *S*_21_ for the first four modes of Resonator 1 as functions of frequency and magnetic flux. The amplitude of *S*_21_ in each subpanel is normalized independently by dividing with the corresponding maximum amplitude. The power in the measurements is approximately −90 dBm at Port 1. The resonance frequencies at half flux quantum are given above the panels, and the simulation parameters are given in Table 1. The measured mode 1 has a lower signal-to-noise ratio compared to the other modes due to unintentional loss near 2.5 GHz in the measurement setup.

The first four resonances of Resonator 1 in Sample A are shown in Fig. 2 as a function of the magnetic flux through the SQUID. The first and the third mode at approximately 2.5 and 7.5 GHz, respectively, do not depend on the flux due to a voltage node in the middle of Resonator 1, i.e., at the coupling capacitor *C*_T_. Thus, these modes are decoupled from those of Resonator 2. In contrast, the second and the fourth mode at 5 and 10 GHz, respectively, show clear flux dependence owing to the changing SQUID inductance, which in turn changes the frequencies of the modes in Resonator 2. If a dissipative mode in Resonator 2 approaches the frequency of a mode in Resonator 1, two distinctive features appear: the resonance in Resonator 1 shifts and broadens owing to the coupling to the dissipative mode. The experimental scattering parameter *S*_21_ is normalized as explained in Methods. The simulation based on the theoretical model (see Methods) shows excellent agreement with experimental data. The slight discrepancy between the experiment and the simulation mainly arises from the uncertainty in the exact values of the parameters given in Table 1.

**Figure 3:**
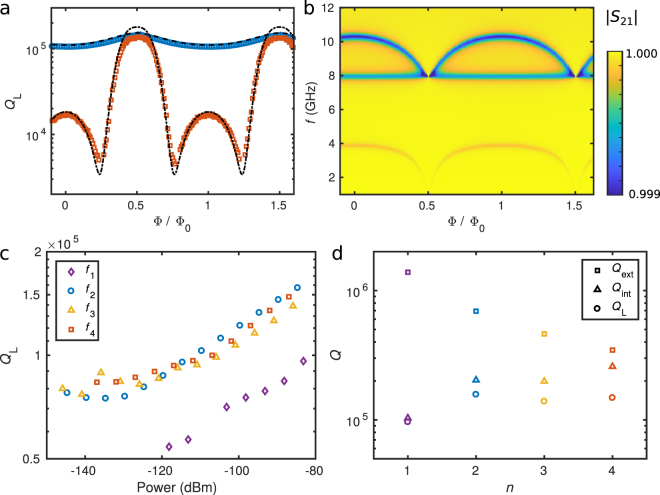
Quality factors of Resonator 1 and resonances of Resonator 2 for Sample A. (**a**) Measured loaded quality factor, *Q*_L_, for mode 2 (blue circles) and for mode 4 (red squares) as functions of the magnetic flux through the SQUID together with the simulated values (dashed line and dash-dotted line, respectively). (**b**) Absolute value of the simulated scattering parameter *S*_21_ of Sample A with only Resonator 2, i.e., at the limit *C*_C_ → ∞. The colour bar is truncated at 0.999 for clarity. (**c**) Measured loaded quality factor, *Q*_L_, of Sample A (markers) for the first four modes as functions of power at Port 1. (**d**) Measured *Q*_L_ of Sample A (circles), predicted external quality factor, *Q*_ext_, (squares) and calculated internal quality factor, *Q*_int_, (triangles) as functions of the mode number. The simulation parameters are given in Table 1. In (**a**), the power at Port 1 is approximately −90 dBm, and in (**d**) −85 dBm. In (**c**) and (**d**), the magnetic flux through the SQUID is Φ/Φ_0_ = 0.5.

The distinctively different flux dependence of modes 2 and 4 in Sample A is clarified by Fig. 3b, which shows the simulated |*S*_21_| with only Resonator 2, i.e., in the limit *C*_C_ → ∞. Resonator 2 has a flux-dependent resonance near 4 GHz, which does not cross the second mode of Resonator 1 at 5 GHz. Nevertheless, it comes sufficiently near 5 GHz, which explains the frequency shift of mode 2 of Resonator 1. In contrast, Resonator 2 has a flux-dependent resonance near 10 GHz, very close to mode 4 of Resonator 1. The resonances intersect which results in dramatic changes in the fourth mode of Resonator 1. The second mode of Resonator 2 near 8 GHz has a current node at the center of the resonator, where the SQUID is located; thus, it is only very weakly dependent on the flux.

Supplementary Fig. 1 shows results similar to those in Fig. 2 for Sample A but for modes 2, 3, and 4 of Sample B. The simulations and experiments are also here in good agreement. However, the simulated mode 2 is substantially narrower than the experimental one. This broadening may arise from an unaccounted mode of the sample holder at a nearby frequency. Furthermore, there is some discrepancy in the phase of mode 4 near integer flux quanta. This discrepancy can be explained by uncertainty in the normalization procedure with very small amplitudes. The first mode is outside the frequency range of the used microwave components, and hence we do not show data for it. For a quantitative comparison of the measured and the simulated resonance frequencies in Samples A and B, Supplementary Fig. 2 shows the frequency shifts of modes 2 and 4 extracted from Fig. 2 and Supplementary Fig. 1. The flux dependence of the modes of Resonator 2 is similar in Sample B to that of Sample A, as shown in Supplementary Fig. 3b. However, the resonances do not intersect at 10 GHz although they are very close to each other.

### Quality factors

We also analyze the quality factors as functions of flux, as shown for Sample A in Fig. 3a, and for Sample B in Supplementary Fig. 3a. The *Q* factors of the second and fourth mode are tunable unlike in the case of the first and third mode. The second mode of Sample A shows only relatively small variation near 10^5^ whereas the fourth mode can be tuned from above 10^5^ down to a few thousand. For Sample B, the flux dependence of the *Q* factor is similar. However, the experimentally determined loaded quality factor, *Q*_L_ for the second mode is substantially lower than the simulated value, i.e., the resonance peak is broader as discussed above. Better agreement between simulation and experiment can be obtained by introducing an additional loss mechanism as described in the caption of Supplementary Fig. 3.

The power dependence of the quality factors is analyzed in Fig. 3c for the four lowest modes in Sample A. The *Q* factors decrease with decreasing power as expected, presumably due to two-level systems in the oxides and on the surfaces and interfaces^33^. The *Q* factors may also be reduced by quasiparticle excitations^34^. Nevertheless, they remain rather close to 10^5^ even at the single-photon level, around −140 dBm. However, relatively high powers enable more accurate measurements of the losses caused by the resistor when the resonators are tuned into the weak coupling regime. Figure 3d shows the experimentally obtained loaded quality factor, *Q*_L_, and the theoretically predicted external quality factor, *Q*_ext_, corresponding to the losses through the coupling capacitors *C*_C_ as functions of the mode number *n*. Furthermore, the internal quality factor, *Q*_int_, corresponding to the internal losses in the system is calculated from the equation ${Q}_{{\rm{int}}}^{-1}={Q}_{{\rm{L}}}^{-1}-{Q}_{{\rm{ext}}}^{-1}$. The internal quality factor slightly increases with the mode number and obtains values near 2.5 × 10^5^. The minimum value of ${Q}_{{\rm{L}}}\lesssim 5\times {10}^{3}$ in Fig. 3a gives also the minimum value for *Q*_int_ since the internal losses of the system dominate when the resistor is strongly coupled to the fourth mode of Resonator 1.

The minimum and maximum *Q*_int_ correspond to photon lifetimes *τ*_int_ = *Q*_int_/*ω*_0_ of 80 ns and 4 *μ*s, respectively, at *ω*_0_ = 2*π* × 10 GHz when other losses are neglected. Furthermore, *Q*_ext_ corresponds to a photon lifetime of 6 *μ*s. These photon lifetimes are long compared to the period of the coherent oscillations between the two resonators at resonance, *τ*_T_ = 30 ns (see Methods). Thus, the internal or external losses of Resonator 1 are not dominating over the coupling strength between the resonators. However, the simulated *Q* factors of the lowest modes of Resonator 2 in Fig. 3b are well below 40 at the zero flux bias and also at Φ/Φ_0_ ≈ 0.2 which corresponds to the crossing of the modes at 10 GHz. They obtain values above 100 only in the range 0.48 < Φ/Φ_0_ < 0.52 due to the ideally diverging SQUID inductance. Thus, the photon lifetime in Resonator 2 is below 0.6 ns at 10 GHz and at the relevant flux point. Consequently, the photons in Resonator 2 are dissipated quickly compared to the period of the coherent oscillations between the resonators, which prevents the formation of well-separated modes hybridized between the resonators. Importantly, Resonator 2 mostly functions as a tunable dissipative environment for Resonator 1, the dissipation of which is limited by the coupling strength between the resonators.

### Simulations with different resistances

**Figure 4:**
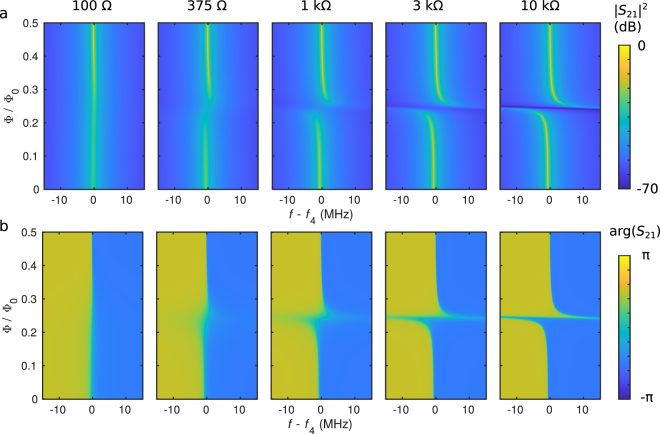
Effect of the termination resistance. Simulated (**a**) amplitude, and (**b**) phase of mode 4 in Sample A as functions of frequency and magnetic flux with different resistance values, *R*, as indicated above the panels. The resonance frequency is *f*_4_ = 9.9 GHz, and the other parameters are given in Table 1.

We also simulate the effect of changing the termination resistance as shown in Fig. 4. The other parameters in the simulations are from Sample A. Note the different frequency range and colour scale compared to Fig. 2. In the case of a 100-Ω termination resistance, there is very little shift in the resonance frequency as a function of the magnetic flux. Nevertheless, the width of the peak varies since the ideal SQUID inductance diverges, *L* → ∞ for Φ/Φ_0_ → 0.5, and therefore, it decouples the resistor from Resonator 1. At even lower resistances near 50 Ω (not shown), the termination is well matched to the characteristic impedance, and hence the description of Resonator 2 as a resonator becomes obscure. Instead, it appears as a broad-band dissipative environment for Resonator 1. With increasing resistance, Resonator 2 obtains well-defined resonances, with zero-flux *Q* factors becoming of the order of 10^3^ at *R* = 10 kΩ. However, the maximum *Q*_L_ in Resonator 1 of 1.8 × 10^5^ does not vary due to the ideally infinite impedance of the SQUID at Φ/Φ_0_ → 0.5. In contrast at zero flux, *Q*_L_ increases from 1.0 × 10^4^ to 1.3 × 10^5^ as the resistance increases from 100 Ω to 10 kΩ. At *R* = 10 kΩ, the two resonators show a clear avoided-crossing feature. There is a continuous crossover from a single modulating resonance at low resistance values to two resonances with an avoided crossing at high resistances. In the experiments, we have *R* = 375 Ω, which results in a single modulating resonance with some avoided-crossing-like features.

## Discussion

We have experimentally demonstrated tunable dissipation in a device consisting of two resonators in very good agreement with our theoretical model. We have studied two samples with slightly different parameters. Both of them allow us to substantially tune the loaded quality factors of the relevant resonances. In addition, the internal quality factor of one of the modes can be tuned from approximately a quarter of a million down to a few thousand. Importantly, we have designed the circuit such that the timescale for the coherent oscillations between the resonators is somewhat shorter than the photon lifetime in Resonator 1 and longer than the photon lifetime in Resonator 2. Therefore, Resonator 2 operates as an efficient dissipative environment for Resonator 1. Note that the spurious internal losses in the system are low as indicated by the high maximum quality factor. Thus, the fabrication of the on-chip resistors is compatible with obtaining high quality factors using our fabrication process. To our knowledge, these are the highest demonstrated quality factors in superconducting resonators with integrated on-chip resistors. In the future, the remaining unwanted losses can be reduced by further improving the process.

Here, we have demonstrated a tunable dissipative environment with a rather specific sample type. Nevertheless, the geometry and parameters can be relatively freely chosen to optimize the heat sink for different applications. For instance, it is possible to modify the losses by changing the resistance and capacitance values. Furthermore, the geometry of the system can be changed in order to obtain different coupling strengths for different modes. In addition, the resistor does not necessarily have to be directly coupled to Resonator 2. Instead, it can be outside the resonator and coupled with a small capacitance and a section of a transmission line. Furthermore, in case the resistance equals to the characteristic impedance of the transmission line resonator, the environment is effectively similar to a transmission line^19^. It is also possible to have an off-chip resistor with a microwave connector. However, an on-chip resistor has several advantages. Firstly, it has a much smaller footprint than a waveguide to an external port. Furthermore, each additional connector per qubit will also increase the total complexity and cost of the circuit as well as hinder the scalability.

Although we consider the resistors only as sources of dissipation here, they may also be engineered to simultaneously function as parts of photon-absorbing normal-metal–insulator–superconductor tunnel junctions^35^, or as quasiparticle traps in superconducting circuits^36–40^. Fast tuning of the quality factors can be obtained by introducing microwave flux bias lines. An upper bound for the speed of the flux tuning is given by the plasma frequency of the SQUID, which is of the order of 30 GHz in our samples. In the future, qubits can be integrated into this system potentially enabling the demonstration of the protocol for fast and accurate initialization^16^. The duration of the protocol depends on the desired fidelity, the accuracy of the flux bias sweep, and the transition rates, and it is predicted to be of the order of a microsecond for high-fidelity initialization with realistic parameters^16^.

## Methods

### Theoretical model and simulations

We analyze the electrical circuit shown in Fig. 1f, which also defines the symbols employed below. The input impedance of Resonator 2 can be obtained from standard microwave circuit analysis^41^, and it is given by

1 $${Z}_{{\rm{r}}2}=\frac{1}{i\omega {C}_{{\rm{T}}}}+\frac{{Z}_{0}\{{Z}_{{\rm{S}}}+{Z}_{0}\,\tanh (\gamma {x}_{2})+\frac{{Z}_{0}[{Z}_{{\rm{term}}}+{Z}_{0}\,\tanh (\gamma {x}_{2})]}{{Z}_{0}+{Z}_{{\rm{term}}}\,\tanh (\gamma {x}_{2})}\}}{{Z}_{0}+{\rm{t}}{\rm{a}}{\rm{n}}{\rm{h}}(\gamma {x}_{2})\{{Z}_{{\rm{S}}}+\frac{{Z}_{0}[{Z}_{{\rm{term}}}+{Z}_{0}\,\tanh (\gamma {x}_{2})]}{{Z}_{0}+{Z}_{{\rm{term}}}\,\tanh (\gamma {x}_{2})}\}},$$

where *Z*_S_ = *iωL* + 2/(*iωC*_L_) is the impedance of the SQUID and the parallel plate capacitors connecting the SQUID to the center conductor, and *Z*_term_ = *R* + 1/(*iωC*_R1_) + 1/(*iωC*_R2_) is the impedance of the terminating resistor and the capacitances connecting it to the center conductor and the ground plane. Here, *ω* = 2*πf* is the angular frequency of the measurement tone, and *γ* is the wave propagation coefficient detailed below. We consider the SQUID as a tunable classical inductor. The inductance of the SQUID as a function of the magnetic flux Φ is ideally given by *L*(Φ) = Φ_0_/[2*πI*_0_|cos(*π*Φ/Φ_0_)|], where *I*_0_ is the maximum supercurrent through the SQUID, and Φ_0_ = *h*/(2*e*) is the magnetic flux quantum. The losses in the SQUID are assumed to be substantially smaller than those induced by the resistor; thus, they are neglected. One could also include a capacitance in parallel with the inductance in the model but it would have only a minor effect as discussed below.

We can calculate the scattering parameter from Port 1 to Port 2 using the ABCD matrix method^41^

2 $${S}_{21}=\frac{2}{A+B/{Z}_{{\rm{L}}}+C{Z}_{{\rm{L}}}+D},$$

where the coefficients are obtained from

3 $$\begin{array}{rcl}(\begin{array}{cc}A & B\\ C & D\end{array}) & = & (\begin{array}{cc}1 & \frac{1}{i\omega {C}_{{\rm{C}}}}\\ 0 & 1\end{array})(\begin{array}{cc}\cosh (\gamma {x}_{1}) & {Z}_{0}\,\sinh (\gamma {x}_{1})\\ \frac{1}{{Z}_{0}}\,\sinh (\gamma {x}_{1}) & \cosh (\gamma {x}_{1})\end{array})\\  &  & (\begin{array}{cc}1 & 0\\ \frac{1}{{Z}_{{\rm{r}}2}} & 1\end{array})(\begin{array}{cc}\cosh (\gamma {x}_{1}) & {Z}_{0}\,\sinh (\gamma {x}_{1})\\ \frac{1}{{Z}_{0}}\,\sinh (\gamma {x}_{1}) & \cosh (\gamma {x}_{1})\end{array})(\begin{array}{cc}1 & \frac{1}{i\omega {C}_{{\rm{C}}}}\\ 0 & 1\end{array})\mathrm{.}\end{array}$$

These equations are solved numerically with Matlab.

The simulation parameters are given in Table 1. We use identical parameters in the simulations for both samples except that the length *x*_2_ is different. The capacitances *C*_C_ and *C*_T_ are based on finite-element-method (FEM) calculations with design geometry and without native oxides, whereas *C*_L_, *C*_R1_, and *C*_R2_ are calculated using parallel-plate-capacitor model by deducing the areas from scanning electron microscope images, and assuming the niobium oxide to have a typical thickness^42^ of 5 nm and relative permittivity^43^ of 6.5. The capacitance per unit length of the coplanar waveguide *C*_l_ is also based on a FEM simulation. The resistance *R* is measured with a dc control sample in a four-probe setup at 10 mK. The test resistor is fabricated in the same process with the actual samples. The effective permittivity of the waveguide *ε*_eff_ is obtained from the nominal widths of the centre conductor and the gap, 10 *μ*m and 5 *μ*m respectively, using an analytical formula^44^. The lengths *x*_1_ and *x*_2_ are design values. The internal quality factor of the first mode of Resonator 1 alone, *Q*_int,1_, is based on measurements of control samples consisting of a single resonator, and it agrees well with the measured first mode of Sample A. The characteristic impedance of the external lines *Z*_L_ has a nominal value, and the characteristic impedance of the resonators *Z*_0_ has a design value in good agreement with the experimental results. The maximum supercurrent through the SQUID *I*_0_ is used as the only fitting parameter since it cannot be directly measured in the actual sample. Nevertheless, the critical current in the actual samples is relatively close to a switching current of approximately 180 nA measured with a dc setup in an essentially similar but separately fabricated control SQUID. Due to noise from a high-temperature environment via the dc lines, the temperature of the control SQUID may be higher than in the actual sample, thus providing an explanation to the difference in the critical current and the switching current. In addition, the difference may well be explained by unintentional differences in the fabrication. We can write the wave propagation coefficient as *γ* = *ω*_1_/(2*Q*_int,1_ *v*_ph_) + *iω*/*v*_ph_, where ${v}_{{\rm{ph}}}=c/(\sqrt{{\varepsilon }_{{\rm{eff}}}})$ is the phase velocity, ${\omega }_{1}\mathrm{/(2}\pi )=c\mathrm{/(4}{x}_{1}\sqrt{{\varepsilon }_{{\rm{eff}}}})$ is the fundamental frequency, and *c* is the speed of light in vacuum.

The loaded quality factor can be defined as *Q*_L_ = *ω*_0_*E*/*P*_loss_, where *ω*_0_ = 2*πf*_0_ is the angular frequency of the resonance, *E* the energy stored in the resonator, and *P*_loss_ = −d*E*/d*t* the power loss. Without input power, the energy in the resonator evolves as a function of time *t* as *E*(*t*) = *E*_0_exp(−*ω*_0_*t*/*Q*_L_), where *E*_0_ is the initial energy. Thus, the photon lifetime is given by *τ*_L_ = *Q*_L_/*ω*_0_, which corresponds to the total losses described by *Q*_L_. Since the number of photons in a resonator *n* depends on the energy as $E=n\hslash {\omega }_{0}$, where $\hslash$ is the reduced Planck constant, and the power loss is bounded from above by the input power *P*_in_ in the steady state, one obtains an upper bound for the photon number as $n < {Q}_{{\rm{L}}}{P}_{{\rm{in}}}/({\omega }_{0}^{2}\hslash )$. Therefore, the average photon number in a 10-GHz resonator is near unity or below if the *Q* factor is 10^5^ and the input power is −140 dBm (c.f. Fig. 3). The external quality factor corresponding to the leakage through the coupling capacitors can be calculated as^5^ ${Q}_{{\rm{ext}}}=2{x}_{1}{C}_{{\rm{l}}}\mathrm{/(4}{Z}_{{\rm{L}}}{\omega }_{0}{C}_{{\rm{C}}}^{2})$. Although *Q*_ext_ calculated with this formula is quite sensitive to errors especially in *C*_C_, it can be considered at least as an order-of-magnitude estimate. The external quality factor is related to the coupling strength describing the external ports, *κ*_ext_ = *ω*_0_/*Q*_ext_ = 2*π* × 30 kHz at *ω*_0_ = 2*π* × 10 GHz. In addition, one can write the photon lifetime without other loss mechanisms as *τ*_ext_ = *Q*_ext_/*ω*_0_ = 1/*κ*_ext_ = 6 *μ*s. The coupling to the external ports can be compared with the coupling strength between the resonators at resonance calculated as^45^ ${g}_{{\rm{T}}}={C}_{{\rm{T}}}{V}_{1}{V}_{2}/\hslash =2\pi \times 10$ MHz, where ${V}_{i}=\sqrt{\hslash {\omega }_{0}\mathrm{/(2}{x}_{i}{C}_{{\rm{l}}})}$, *i* = 1,2, and *ω*_0_ = 2*π* × 10 GHz. Furthermore, the period for coherent oscillations between the resonators can be written as^3^ *τ*_T_ = *π*/*g*_T_ = 30 ns, where we have neglected dissipation.

The junction capacitance can be estimated using a parallel-plate model with an approximate aluminium oxide thickness of 2 nm, a junction area of 0.25 μm^2^ estimated from micrographs, and a typical relative permittivity^46^ of 8.2, which yield 10 fF per junction. At zero flux and 5 GHz and 10 GHz, the inductive reactance of the SQUID is 40 Ω and 80 Ω, whereas the capacitor consisting of two junctions in parallel has a reactance of 2 kΩ and 0.9 kΩ, respectively. If included in the model, the capacitive shunt of the inductance could result in a very small change of the scattering parameter *S*_21_ at Φ/Φ_0_ ≈ 0.5 where the inductance ideally diverges. The change is small owing to the weak coupling of the resonators. Consequently, we do not include it in the model. Thus, the effect of the capacitance is effectively included in that of the inductance, which depends on the fitting parameter *I*_0_. The plasma frequency of the SQUID can be obtained as ${\omega }_{{\rm{p}}}\mathrm{/(2}\pi )=\mathrm{1/(2}\pi \sqrt{LC})$, where *L* is the inductance and *C* the capacitance of the junctions.

### Sample fabrication

Samples A and B are fabricated in the same process. The actual samples as well as the control samples are fabricated on 100-mm Si wafers. First, native SiO_2_ is removed with ion beam etching, and 200 nm of Nb is sputtered onto the wafer without breaking the vacuum.

Second, the large patterns are defined using standard optical lithography. The optical lithography begins with hexamethyldisilazane priming, followed by spin coating the resist AZ5214E at 4000 rpm. The resist is exposed using a mask aligner in a hard-contact mode, and the exposed resist is removed with the developer AZ351B. In order to obtain a positive profile for the Nb edges, we apply a reflow bake at 140 °C before reactive ion etching. Once the large patterns are ready, we pre-dice the wafer half way from the back side.

In the third step, the nanostructures are defined using electron beam lithography (EBL). After thorough cleaning of the wafer with a plasma stripper, a resist for EBL is spin-coated to the wafer. The EBL resist consists of two layers: poly(methyl methacrylate) with 4% of anisole, and poly[(methyl methacrylate)-co-(methacrylic acid)] with 11% of ethyl lactate. We fabricate all the nanostructures in a single EBL write. For the development, we use a 1:3 solution of methyl isobutyl ketone and isopropanol. The metallization for the nanostructures is carried out with an electron beam evaporator in two steps. First, the Cu resistor is evaporated followed by the evaporation of the SQUID. We evaporate Cu only on the area in the vicinity of the resistor on the chip and keep the rest of the chip covered by a metal mask. Subsequently, we cover the resistor and evaporate the Al structures. The SQUID consists of two Al layers evaporated at two angles (±15°). The oxide layer for the Josephson junctions is obtained by oxidizing Al *in situ* in the evaporation chamber at 1 mbar of O_2_ for 5 min. The lift-off process is carried out in acetone followed by cleaning with isopropanol. The Cu resistor has a width of 250 nm, thickness of 30 nm, and length of 90 *μ*m. The SQUID consists of two layers of Al with thicknesses of 40 nm each, and it has a loop area of approximately 50 *μ*m^2^.

### Measurement setup

The measurement setup is shown in Supplementary Fig. 4. The measurements are carried out in a dry dilution refrigerator with a base temperature of approximately 10 mK, and the scattering parameters are measured with a vector network analyzer (VNA). We control the magnetic flux through the SQUID using an external coil attached to the sample holder, and the current through the coil is generated with a source-measure unit (SMU). The sample is wire-bonded to a printed circuit board shielded by a sample holder that is fabricated out of Au-plated Cu. The sample holder is placed inside a magnetic shield to mitigate magnetic-field noise.

### Normalization of scattering parameters

All raw experimental scattering parameters are normalized. First, the winding of the phase as a function of frequency is cancelled for convenience by multiplying *S*_21_ with exp(*i*2*πfτ*) where $\tau \approx 50$ ns. Second, the circle in the complex plane drawn by *S*_21_ when the frequency is swept through the resonance is shifted and rotated to its canonical position, where the circle intersects the origin and the maximum amplitude lies on the positive *x* axis^47^. Any uncertainty in this shift causes relatively large errors near origin; hence, we use linear scale for experimental data as it emphasizes the large amplitudes with smaller relative error. Consequently, one can extract the *Q* factor using the phase–frequency fitting method discussed in ref.^47^. In addition to the experimental *Q* factors, we use the same method for obtaining the *Q* factor also from the simulations, except that the very low *Q* factor of Resonator 2 is obtained from the width of the dip. In order to exclude uncertainty related to the cable losses, we normalize *S*_21_ by dividing it with ${{\rm{m}}{\rm{a}}{\rm{x}}}_{f,{\rm{\Phi }}}|{S}_{21}|$ separately for each mode. The magnetic flux is extracted from the periodicity of the of modes 2 and 4, and there can be an irrelevant offset of an integer number of flux quanta. One flux quantum corresponds to an electric current of approximately 2 mA in the coil used.

### Data availability

The data are available upon request from the authors.

## Electronic supplementary material

Supplementary information
