# Supplementary material for: Flux-tunable heat sink for quantum electric circuits
Source: Sci Rep. 2018 Apr 20;8:6325. doi: 10.1038/s41598-018-24449-1 (PMC5910410; doi:10.1038/s41598-018-24449-1)
Supplement: Supplementary file 1 — Supplementary information [file 41598_2018_24449_MOESM1_ESM.pdf]

Supplementary information

Flux-tunable heat sink for quantum electric circuits

M. Partanen[1,*], K. Y. Tan[1], S. Masuda[1], J. Govenius[1], R. E. Lake[1,2], M. Jenei[1], L. Grönberg[3], J. Hassel[3], S. Simbierowicz[3], V. Vesterinen[1,3], J. Tuorila[1,4,5], T. Ala-Nissila[3,6,7], and M. Möttönen[1,†]

[1]QCD Labs, QTF Centre of Excellence, Department of Applied Physics, Aalto University, P.O. Box 13500, FI-00076 Aalto, Finland
[2]National Institute of Standards and Technology, Boulder, Colorado 80305, USA
[3]VTT Technical Research Centre of Finland, P.O. Box 1000, FI-02044 VTT, Finland
[4]MSP group, QTF Centre of Excellence, Department of Applied Physics, Aalto University, P.O. Box 13500, FI-00076 Aalto, Finland
[5]Nano and Molecular Systems Research Unit, University of Oulu, P.O. Box 3000, FI-90014 Oulu, Finland
[6]Departments of Mathematical Sciences and Physics, Loughborough University, Loughborough, Leicestershire LE11 3TU, United Kingdom
[7]Department of Physics, Brown University, Box 1843, Providence, Rhode Island, 02912-1843, USA
[*]matti.t.partanen@aalto.fi
[†]mikko.mottonen@aalto.fi

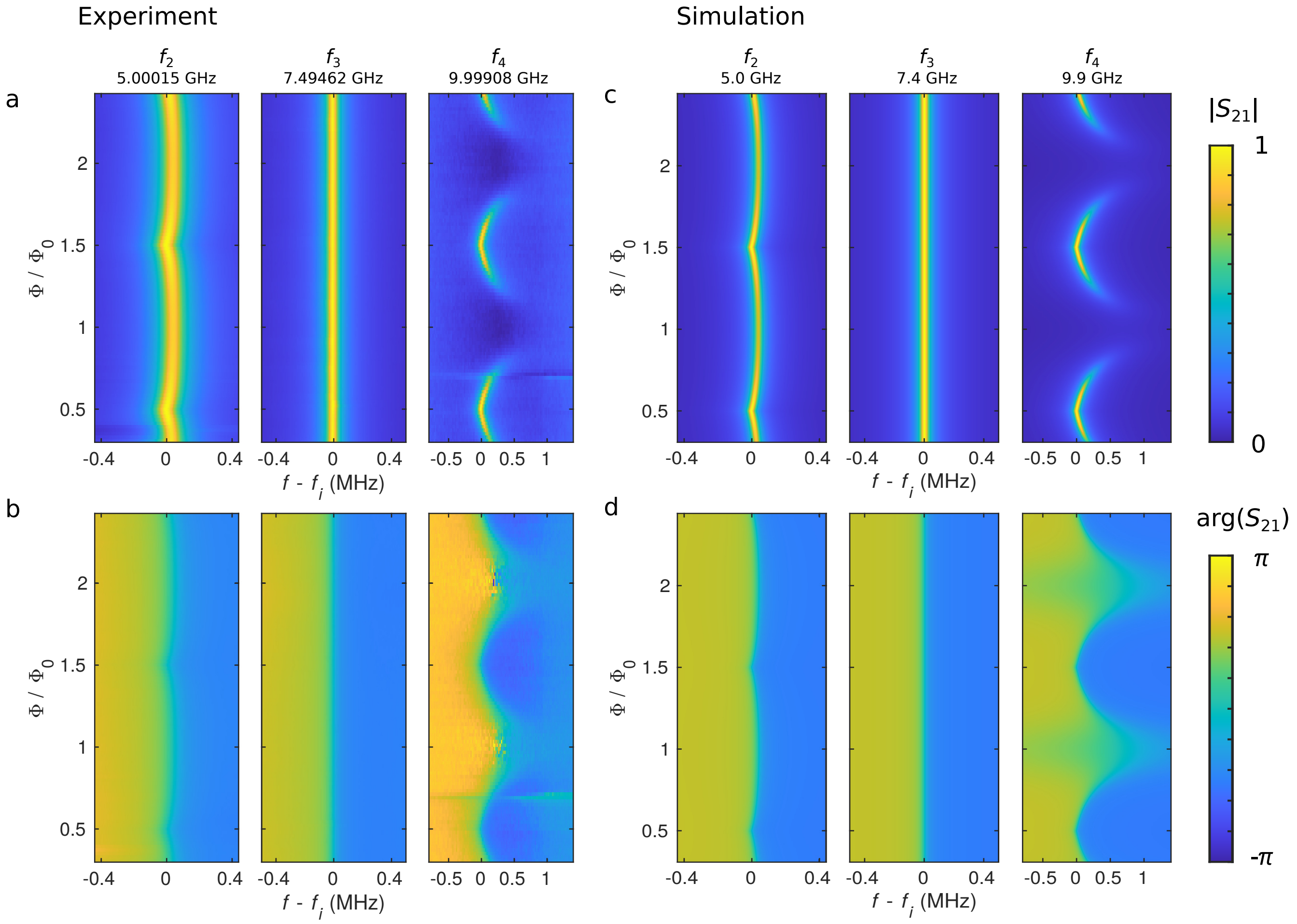

Supplementary Figure 1. Resonances of Sample B. (a,b) Experimental and (c,d) computational scattering parameter S_{21} for the modes 2,3, and 4 of Resonator 1 as functions of frequency and magnetic flux. (a,c) Normalized amplitude of S_{21}. Each panel is normalized separately by dividing with the corresponding maximum amplitude. (b,d) Phase of S_{21}. The resonance frequencies are given above the panels, and the simulation parameters are given in Table 1. The power in the experiments is approximately -90 dBm at Port 1.

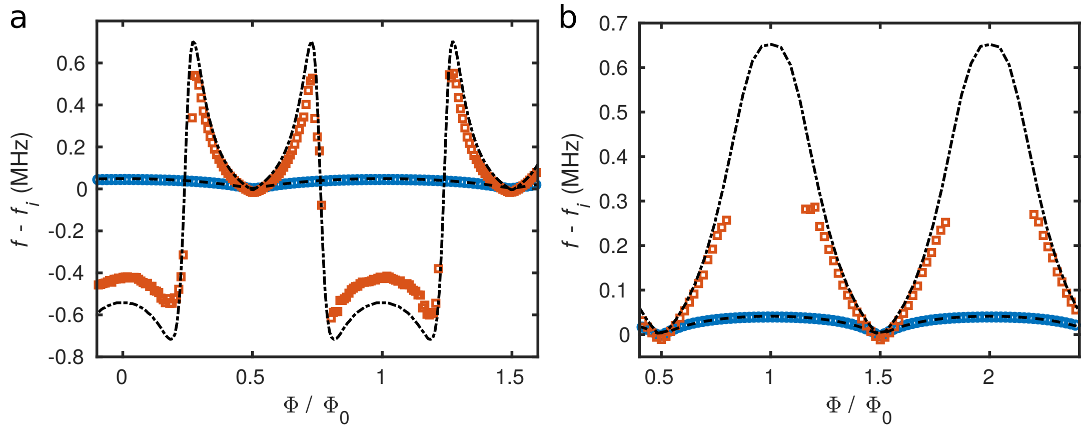

Supplementary Figure 2. Resonance frequency shifts from the magnetic flux point $\Phi/\Phi_0 = 0.5$. (a) Measured frequency shifts of modes 2 (blue circles) and 4 (red squares) together with the corresponding simulations for modes 2 (dashed line) and 4 (dash-dotted line) of Sample A as functions of the magnetic flux. (b) As (a) but for Sample B. The simulation parameters are given in Table 1. In Sample A the measured frequencies are $f_2 = 4.96677$ GHz and $f_4 = 9.92908$ GHz, and and in Sample B $f_2 = 5.00015$ GHz and $f_4 = 9.99908$ GHz.

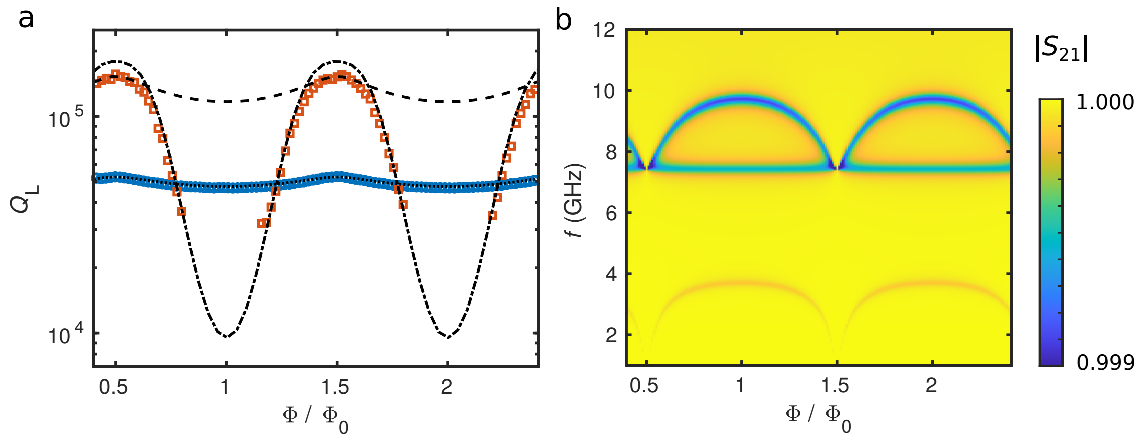

Supplementary Figure 3. Quality factors of Resonator 1 and resonances of Resonator 2 for Sample B. (a) Measured loaded quality factor, Q_{L}, for mode 2 (blue circles), and for mode 4 (red squares) as functions of the magnetic flux through the SQUID together with the simulated values (dashed line and dash-dotted line, respectively). The dotted line on top of the blue circles shows the simulation for the mode 2 with an additional spurious loss mechanism with a flux-independent quality factor of $Q_{\mathrm{sp}} = 8 \times 10^4$ in addition to the simulated quality factor, $Q_{\mathrm{L,si}}$, yielding $Q_{\mathrm{L}}^{-1} = Q_{\mathrm{L,si}}^{-1} + Q_{\mathrm{sp}}^{-1}$ with a better match with the experimental data. The applied power is approximately -90 dBm at Port 1. (b) Absolute value of the simulated scattering parameter S_{21} with only Resonator 2, i.e., at the limit $C_{\mathrm{C}} \to \infty$. The colour bar is truncated at 0.999 for clarity. The simulation parameters are given in Table 1.

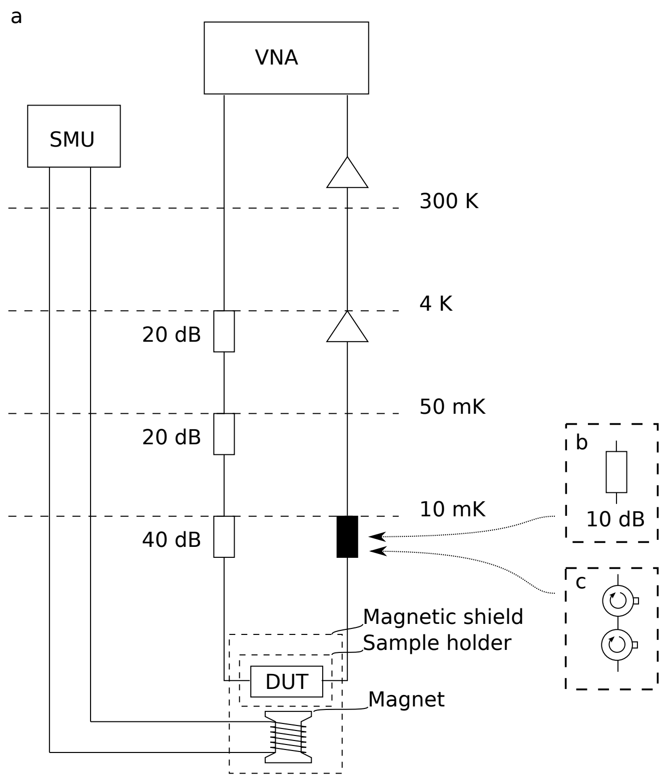

Supplementary Figure 4. Measurement setup. (a) Overview of the measurement setup with different temperature stages of the cryostat indicated. The scattering parameters of the device under test (DUT) are measured with a vector network analyzer (VNA), and the magnetic flux through the SQUID is produced with a bias current generated by a source measure unit (SMU). (b) For Sample A, a 10-dB attenuator is employed after the sample in the position of the black box to prevent amplifier noise from entering the sample. (c) For Sample B, two isolators are used instead.
